# Integrating bioenergetics and conservation biology: thermal sensitivity of digestive performance in Eastern Collared Lizards (*Crotaphytus collaris*) may affect population persistence

**DOI:** 10.1093/conphys/coaa018

**Published:** 2020-09-08

**Authors:** Casey L Brewster, Jason Ortega, Steven J Beaupre

**Affiliations:** Department of Biological Sciences, University of Arkansas, Fayetteville, AR 72701, USA

**Keywords:** feeding rate, metabolizable energy, net assimilated energy, passage time, thermal performance

## Abstract

Information on bioenergetics can provide valuable insight into the ecology, life history and population dynamics of organisms. For ectothermic animals, thermal sensitivity of digestion is an important determinant of net assimilated energy budgets. A recent study in the Ozark Mountains indicated that eastern collared lizards (*Crotaphytus collaris*) restricted to encroached glades (characterized by woody vegetation encroachment) experience reduced environmental heat loads and have reduced age-specific growth and reproductive rates compared to populations in intact glades. To assess the potential impact of reduced body temperatures on assimilation rates of *C. collaris* in encroached glades, we conducted feeding trials across four temperature treatments (28, 31, 34 and 37°C). We tested for temperature effects on voluntary feeding rates, passage times, apparent assimilated energy (AE) and metabolizable energy (ME). Passage times decreased and voluntary feeding rates increased significantly with increasing temperature. Consumption explained the majority of variance in AE and ME, followed by the effect of temperature treatments. Using data on voluntary feeding rates, passage times and ME as a function of temperature, we estimated over a 10-fold increase in predicted daily assimilated energy across temperature treatments (28°C = 0.58 kJ/day, 31°C = 1.20 kJ/day, 34°C = 4.30 kJ/day, 37°C = 7.95 kJ/day). Thus, lower heat loads in encroached glades may cause reduced body temperature and result in restricted energy assimilation rates. Our study provides a novel approach to the integration of bioenergetics and conservation and shows the efficacy of using information on digestive performance to investigate underlying mechanisms in a conservation context.

## Introduction

The integration of physiology and conservation is an emerging field (Wikelski and Cooke 2006) that has provided valuable perspective in addressing negative impacts on biodiversity (Madliger *et al*. 2016). Recently, researchers have expanded the ‘conservation physiology toolbox’ (Madliger *et al*. 2018), greatly improving our ability to address underlying mechanisms associated with environmental change. The importance of bioenergetics to conservation physiology has been recently emphasized (Tracy *et al*. 2006; Mochnacz *et al*. 2017; Leo *et al*. 2018; Madliger *et al*. 2018). Information from bioenergetics can provide valuable insight into the ecology, life history and population dynamics of species (Porter and Tracy 1983; Dunham *et al*. 1989; Roff 1991; Adolph and Porter 1993; Dunham and Overall 1994; Anger 1995; Beaupre 2002), which in turn, can be used to address conservation-based questions (Tracy *et al*. 2006; Cerino *et al*. 2013; Marras *et al*. 2015; Steell *et al*. 2019). From a physiological standpoint, the thermal dependence of digestive performance strongly influences bioenergetics of free-ranging animals (Congdon *et al*. 1982; Angilletta 2001; Beaupre 2002; Pang *et al*. 2011; Beaupre and Zaidan 2013). Digestive physiology determines the net assimilated energy available for competing functions of growth, reproduction, maintenance and storage (Congdon *et al*. 1982; Niewiarowski 2001)—which directly affects fitness (Dunham and Overall 1994). Thus, information on digestive performance represents a valuable physiological tool that can be integrated into a conservation context.

In the Ozark Mountains of Arkansas and Missouri, the eastern collared lizard (*Crotaphytus collaris*) has been listed as a species of special concern (state level listing of S2 and S4 respectively) as a result of extensive population declines (Templeton *et al*. 2001; Grimsley 2012). Previous research on *C. collaris* in northern Arkansas has documented reduced age-specific growth and reproductive rates in populations inhabiting encroached glades (e.g. glade habitats characterized by dense woody vegetation cover; Brewster *et al*. 2018). Encroached glades had increased shade cover compared to intact glades (e.g. glade habitats characterized by low woody vegetation density), reducing daily environmental heat loads in those habitats (Porter and Gates 1969; Huang *et al*. 2014). A reduction in environmental heat loads implies that *C. collaris* in degraded habitats may experience lower body temperatures (*Tb*s). Since most physiological functions are thermally dependent (Huey 1982; Peterson *et al*. 1993), a reduction in *Tb* could lead to a decline in digestive processing rates (van Marken Lichtenbelt 1997; Grant and Dunham 1990), ultimately causing reduced growth and reproduction of *C. collaris* in encroached glades. Thus, understanding the effect of *Tb* on the digestive performance of *C. collaris* is an important component to identifying potential explanatory mechanisms resulting in reduced reproductive rates and population declines of this species in Arkansas.

The objective of this study was to determine the thermal sensitivity of digestive performance in *C. collaris*. Specifically, we tested for differences in (i) passage time (defined as time to first and last presence of a marked meal in faeces), (ii) apparent assimilated energy (AE; energy consumed—energy lost in faeces), (iii) metabolizable energy (ME; energy consumed—energy lost in faeces—energy lost in uric acid) and (iv) voluntary feeding rates of *C. collaris* from two populations across four temperature trials (28, 31, 34 and 37°C). We used these data to estimate the potential effect of reduced *Tb*s on energy assimilation of *C. collaris*.

## Methods

### Captive maintenance

We captured 50 wild *C. collaris* individuals from two rock quarries in northern Arkansas and transported them to the University of Arkansas. Individuals ranged in body mass from 11.2 to 34.0 g and included 22 males and 28 females. Animals were housed individually in clear plastic terraria (38 L) with butcher paper subflooring and a single plastic hide box (~0.5 L). Terraria were placed on metal shelving inside of a temperature controlled walk-in environmental chamber with 12:12 photoperiod. The inside of terraria was spritzed daily with fresh water, and animals were fed crickets (*Acheta domestica*) every other day throughout the course of the study. Including acclimatization (3 days) and experimental trails (14 days), all animals were captured, studied and returned healthy to their point of capture in less than 20 days.

### Experimental design: voluntary feeding rates

Individuals were randomly assigned to one of six temperature treatments: 21, 25, 28, 31, 34 and 37°C, but subject to the constraint that sex and population of origin were equally represented in each treatment. Thermal treatments were chosen that fall within the range of *Tb*s observed in *C. collaris* during the activity season (20–42.6°C; CLB unpublished dissertation). For the 28–37 °C treatments, we assigned 10 individuals (4 males: 6 females at 31 and 37°C; 5:5 at 28 and 34°C) to each treatment group, with an even ratio of population of origin. We used five individuals per treatment group in the 21 and 25°C (2 males, 3 females and 2:3 ratio of population of origin). We placed six live adult crickets in each individuals’ terrarium and recorded the number of crickets consumed after 2 h. For our metric of voluntary feeding rates, we used the number of crickets consumed in 2 h as a function of temperature. Crickets used for feeding trials were obtained from a commercial dealer (Fluker’s Cricket Farms, Baton Rouge, LO).

### Experimental design: energy extraction and passage time

Temperature treatments for energy extraction and passage time (28–37°C) were chosen based on results from the voluntary feeding trials, which suggested *C. collaris* refused to eat at *Tb*s below 28°C. Thus, after voluntary feeding trials, individuals from the 21 and 25 °C treatment groups were released healthy to their population of origin within 5 days of capture. The remaining 40 individuals from the 28–37°C trials were used, in their respective temperature treatments, for the digestion and passage time experiments.

Animals were fed a live cricket meal (two to eight crickets) equal to 5.5% (±0.25%) of their body mass every other day, starting on the day after capture. We weighed crickets and lizards to the nearest 0.001 g using an analytical scale. Beginning with the second feeding event, we fed lizards a marked meal at every other feeding event. Previous research suggests that absorptive state and meal frequency can affect digestive processing estimates in lizards (Windell and Sarokon 1976; Skoczylas 1978; Waldschmidt *et al*. 1986). Thus, to reduce the potential effect of variation in meal frequency in our estimates, we did not use any faeces corresponding to the first fed meal. Meals were marked by injecting ~ 0.02 mL of inert UV fluorescent powder slurry (Slice of The Moon, Toronto, Canada) at a concentration of 0.015 g mL^−1^ and an estimated energy density of 23.6 kJ g^−1^ into the body of each cricket used to make up a single meal. We corrected for the energy content attributed to marker powder (~0.76%) by subtracting the caloric value of marker from the caloric value of marked faeces. We alternated the colour powder used (green, pink, blue or orange) at every marked feeding event to improve delineation of specific meals for quantifying passage times.

Lizards were fed by holding the individual in one hand and presenting a cricket (using a pair of plastic forceps) in front of their face with the opposite hand. Although biting responses by *C. collaris* appeared to be more defensive than a true feeding response, in the majority of trials lizards willingly ate all of their cricket meals using this technique. In cases that animals refused to bite the cricket (this was more common in the 28°C temperature treatment), another researcher would gently tap the lizard on the snout (thus invoking a defensive biting response) while the other researcher quickly placed the cricket into the animal’s mouth. Using this feeding strategy, we were able to feed every subject their allotted meal on every feeding event (eight total feeding events). Previous research suggests that force-feeding *C. collaris* intact meals directly into the oesophagus (precluding normal mastication) can have negative impacts on digestive estimates (Ruppert 1980). The feeding protocol we used in this study allowed all lizards (regardless of temperature treatment) to fully masticate every cricket fed to them.

During the experiment, terraria were checked for faeces and uric acid every 3 from 7:00 am to 7:00 pm. At each check, we removed all waste material, recorded the date and time of the waste event, and checked faeces for the presence of fluorescent marker using a black light. Faeces and uric acid were then bagged separately in an individually marked Whirl-Pak® (Nasco; with animal ID, date, time and wet mass of sample). We also collected five cricket samples (four to six individual crickets per sample) representing a random subsample of crickets fed to *C. collaris* in the study to estimate the average water content and energy density of food consumed by individuals. We stored samples in a freezer until they could be processed at a later date. All samples were later lyophilized for 24 h. We recorded the dry mass of all samples (to the nearest 1 mg), and then combined samples (2–12 separate faecal samples per individual) to meet the minimum sample mass for calorimetry equipment at the University of Arkansas (250 mg). Samples were homogenized prior to analyses and energy densities were estimated using bomb calorimetry by an automated calorimeter at the Central Analytical Lab at the University of Arkansas.

We obtained several response variables from feeding trials including passage time (time in hours to first and last observed presence of a marked meal, TFM and TLM, respectively), total energy consumed (C in kJ), total energy in faeces (F in kJ) and total energy in uric acid (U in kJ). We did not standardize the number of feeding events used to estimate energy lost in F and U. Instead, we used the maximum number of feeding events for each individual that we could confidently assign known feeding events to, based on the presence/absence of fluorescent markers in their faeces.

### Statistical analyses

We used mixed linear ANCOVA implemented with the nlme package (Bates *et al*. 2015) in R (v3.1.3, R Core Team, 2015), with temperature as the main effect in all of our comparisons. We designated population (two populations) as a random factor, with sex as a fixed effect and body size (initial body mass) as a covariate. For AE, ME, F and U comparisons, we included consumption as a second covariate (Raubenheimer and Simpson 1994; Beaupre and Dunham 1995; Raubenheimer 1995). For passage time, we obtained one to three estimates of TFM and TLM for each individual, and we used all obtained estimates for statistical comparisons. Thus, for passage time, we included individual as a random factor in the ANCOVA models. We included individual meal size (dry mass of crickets) as a second covariate for passage time. We designated model covariance structure based on minimum AICc score (Bozdogan 1987). In all analyses, assumptions of normality and homogeneity of slopes were met.

## Results

### Voluntary feeding rates

**Figure 1:**
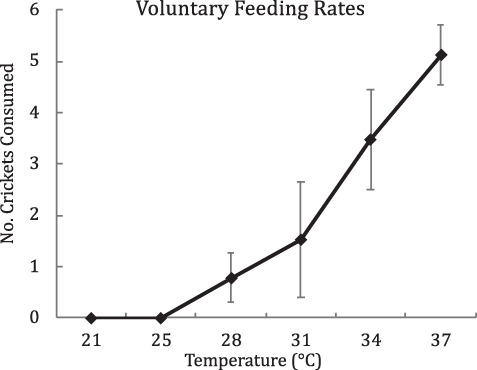
Adjusted (mixed model ANCOVA) means (28–37°C) and raw means (21 and 25°C) of the number of crickets consumed voluntarily in 2 h across temperature treatments. All individuals were offered six live crickets. Error bars = 95% CI. 28–37 °C, *P* < 0.0001

All individuals in the 21 and 25°C voluntary feeding trials ate zero crickets (Fig. 1). Thus, we only made statistical comparisons on the remaining 28–37°C temperature trials. We found a strong effect of temperature on voluntary feeding rates in *C. collaris* across the 28–37°C temperature trials (*F*_3/32_ = 21.85, *P* < 0.0001; Fig. 1). Mixed-model ANCOVA found no significant effect of sex or body mass (covariate). Random effects (population) accounted for 4.5% of the total variance.

### Passage times

**Figure 2:**
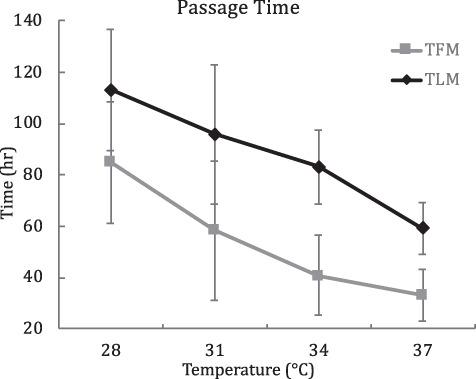
Adjusted (mixed model ANCOVA) means of passage time (h) across four temperature treatments. Passage time defined as time to first mark observed (TFM) and time to last mark observed (TLM). Error bars = ±95% CI. *P* < 0.001 for TFM and TLM

The best model (based on AICc) for passage time comparisons included individual (without population) as a random effect. We converted wet mass of crickets consumed to dry mass using the equation: dry mass = 0.404*wet mass − 0.303 (*n* = 21, *r*^2^ = 0.95, *P* < 0.001). Temperature had a strong effect on passage time for both TFM (*F*_3/34_ = 17.48, *P* < 0.0001) and TLM (*F*_3/34_ = 17.80, *P* < 0.0001; Fig. 2). Mixed-model ANCOVA found no significant effect of sex, meal size (covariate) or individual body mass (covariate) on TFM and TLM. Random effects (individual) accounted for 1.5% (TFM) and 1.1% (TLM) of total variance.

**Figure 3:**
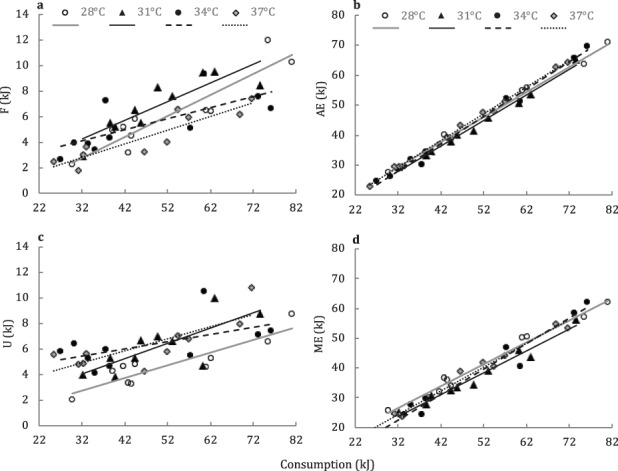
**a–d** Linear relationship between consumption and energy lost in faeces (F), assimilated energy (AE), energy lost in uric acid (U) and metabolizable energy (ME) across four temperature treatments. Raw data points of individuals, and linear regression lines by temperature treatment. Linear regression analysis for each line provided in Appendix 1

### Apparent assimilated energy

**Figure 4:**
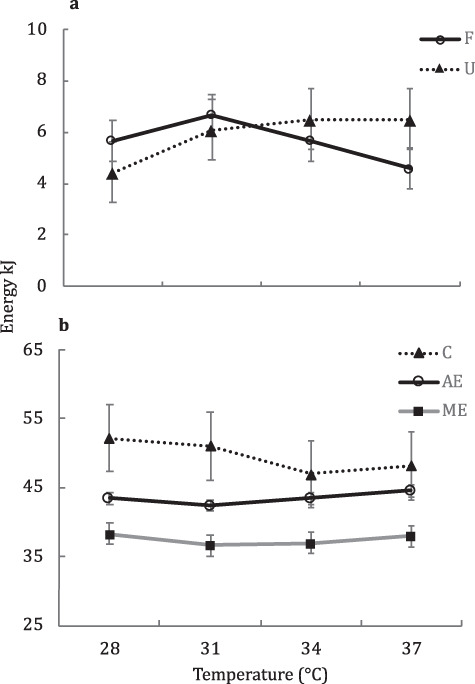
**a–b**: Covariate (consumption) adjusted means (mixed model ANCOVA) of energy utilization in *C. collaris* across four temperature treatments. (a) Energy lost in F (faeces) and U (uric acid). F, *P* < 0.001; U, *P* = 0.018. C (covariate) *P* < 0.001 for F and U. (b) C (consumption; raw means), AE (C–F) and ME (C–F–U). C, *P* = 0.15; AE, *P* < 0.001; ME, *P* = 0.057. C (covariate) *P* < 0.001 for AE and ME. Error bars = 2 SE

For all individuals, we were able to assign a minimum of three (two marked and one unmarked) and a maximum of five (three marked and two unmarked) feeding events in which to estimate C, F and U (grand mean of 4.1 meals, SE = 0.080). The number of meals used for analyses did not differ significantly among temperature treatments (*F*_3,36_ = 1.333, *P* = 0.279). The average energy density of crickets was 22.6 kJ g^−1^ dry mass (SE = 0.60; *n* = 5 samples), and the average energy density of faeces was 18.41 kJ g^−1^ dry mass (SE = 0.35; *n* = 40 samples). The average total energy consumed was 49.64 kJ (SE = 2.48) per individual and did not differ significantly across treatments (standard ANCOVA with sex and temperature as fixed effects, and body mass as a covariate; *F*_3/34_ = 1.895, *P* = 0.149). Energy lost in F was unaffected by body mass (covariate) or sex but was strongly affected by consumption (*P* < 0.0001; Fig. 3a). The effect of temperature on F was also significant (*F*_3/33_ = 6.926, *P* < 0.001; Fig. 4a). Random effect of population accounted for less than 1% of the total variance.

Apparent assimilated energy was unaffected by body mass (covariate) or sex but strongly affected by consumption (*P* < 0.0001; Fig. 3b). The effect of temperature on AE was also significant (*F*_3/33_ = 4.71, *P* = 0.0076; Fig. 4b). Population (random effect) accounted for less than 1% of the total variance. Linear regression of the effect of C on AE across all temperature treatments was: AE = 0.874C + 0.481 (Adj. *r*^2^ = 0.98, *F*_1,38_ = 3443, *P* < 0.0001, SE slope = 0.015, SE intercept = 0.767). Thus, apparent assimilation efficiency was ~87% across temperature treatments. We provide a summary table of linear-regression estimates of temperature-specific AE and F in Appendix 1.

### Metabolizable energy

The average energy density of U was 10.80 kJ g^−1^ dry mass (SE = 0.127, *n* = 20 samples). Energy lost in U was unaffected by body mass (covariate) or sex but was strongly affected by consumption (covariate; *P* < 0.0001; Fig. 3c). The effect of temperature on U was significant (*F*_3/33_ = 3.89, *P* = 0.0173; Fig. 4a). Random effect of population accounted for 6.3% of the total variance.

For ME, we found no significant effect of sex or body mass. Mixed-model ANCOVA revealed a significant positive effect of consumption (*P* < 0.0001; Fig. 3d). Temperature had a statistically significant effect (*F*_3/33_ = 2.933, *P* = 0.0478; Fig. 4b) on ME. Population accounted for 4.9% of the total variance in ME. The relationship between C and ME across all temperature treatments was: ME = 0.793C–1.4621 (Adj. *r*^2^ = 0.96, *F*_1/38_ = 1072, *P* < 0.0001; SE slope = 0.024, SE intercept = 0.124). Thus, metabolizable energy efficiency was ~79% across temperature treatments. We provide a summary table of linear-regression estimates of temperature-specific ME and U in Appendix 1.

## Discussion

The goal of this study was to investigate the potential impact that increased shade and reduced environmental heat loads (characteristics of encroached glades; Brewster *et al*. 2018) may have on energy assimilation rates of *C. collaris*. Specifically, our objective was to assess the thermal sensitivity of digestive performance by testing for a temperature effect on voluntary feeding rates, passage time, apparent assimilated energy and metabolizable energy in *C. collaris*. First, we found a strong effect of temperature on the voluntary feeding rates of *C. collaris* (Fig. 1). Individuals consumed no crickets at *Tb*s below 28°C, few crickets between 28 and 31°C, but consumed most or all crickets offered to them at 34 and 37°C. A recent study (Brewster and Beaupre 2019) suggests minimum voluntary active *Tb* of *C. collaris* in northern Arkansas is 31.2°C (central 99% of surface-active *Tb* = 31.2–42.6°C). Thus, it is not overly surprising that *C. collaris* in this study consumed few crickets at *Tb*s below 31°C. Next, we found a strong effect of *Tb* on passage rates of *C. collaris*. Passage times of animals at 28°C (TFM = 84.9 h, SE = 12.17; TLM = 113.3 h, SE = 12.09, *n* = 20) were approximately twice as long as animals at 37°C (TFM = 33.2 h, SE = 5.26; TLM = 59.3 h, SE = 5.12, *n* = 29; Fig. 2).

In all of our comparisons of energy extraction (F, U, AE and ME), we found a significant effect of consumption, but no effect of body mass. Although each meal size fed to subjects was based on the body mass of individuals (5.5%, ±0.25%), the number of meals used to estimate energy extraction varied among individuals (three to five meals). Thus, consumption was decoupled from body mass in our analyses and allowed differentiation between the effect of consumption (statistically significant) *versus* the effect of body mass (statistically non-significant) on energy extraction estimates. Additionally, all of our models suggested no effect of sex and that population explained a relatively small fraction of total variance (≤6.3%). Thus, data on energy extraction were explained primarily by consumption (Fig. 3) and, to a lesser extent, temperature (Fig. 4). We found a statistically significant effect of *Tb* on AE (C–F), as energy lost to F appeared to decrease nonlinearly across temperature (Fig. 4a and b). Conversely, energy lost to U appeared to increase nonlinearly across temperature (Fig. 4a), presumably as a result of increased metabolic rate with temperature (Coulson and Hernandez 1964; Beaupre *et al*. 1993; Hume 2005). Thus, our results imply that although AE appears to increase slightly across temperature treatments, this trend is lost by the increasing of uric acid production, which partially explains the relatively flat response in ME across *Tb* treatments (Fig. 4). We interpret these finding to suggest that consumption explains the majority of variance in *C. collaris* energy extraction indices, followed by the effect of temperature treatments over the 28–37°C *Tb* range used in the study.

All of our findings indicate that over the range of 28–37°C, digestive performance of *C. collaris* is sensitive to temperature from a statistical standpoint. The thermal dependence of passage time and feeding rate appears to be ubiquitous in lizards (Van Damme *et al*. 1991; Beaupre *et al*. 1993; Chen *et al*. 2003; McConnachie and Alexander 2004). On the other hand, the thermal dependence of energy extraction indices in lizards appears to be species dependent, with some showing no effect of temperature (Christian 1986; van Marken Lichtenbelt 1992; Du *et al*. 2000; Chen *et al*. 2003), and others showing significant thermal dependence (Waldschmidt *et al*. 1986; Troyer 1987; McKinnon and Alexander 1999; Pafilis *et al*. 2006). However, the more important question here is: are the observed differences in digestive performances biologically meaningful for *C. collaris*? Indeed, the ultimate goal of this study was to determine the potential effects of reduced *Tb*s in *C. collaris* on energy assimilation rates in northern Arkansas. Thus, to address the biological implications of our findings we estimated two indices for daily assimilation rates as a function of temperature: ‘standardized’ ME rates and ‘realized’ ME rates. We define standardized ME rates as the predicted metabolizable energy assimilated per day assuming a standard consumption rate (grand mean of daily consumption rate across energy extraction treatments) and the measured effect of temperature on ME and passage times (ME/TFM). Table 1 provides the results of the standardized ME rate comparisons and shows the substantial effect of temperature on daily metabolizable energy rates. Results in Table 1 also show the strong impact that passage rates (TFM as a function of *Tb*) have on energy assimilation rates, relative to ME (as a function of *Tb*). However, our comparisons of standardized ME rates assume that *C. collaris* consumption is insensitive to *Tb*—and our data suggest consumption is highly sensitive to *Tb* (Fig. 1). To account for *Tb* effects on consumptions rates, we define a second index—realized ME rates, which is the average metabolizable energy assimilated per day using the mean consumption rates from the voluntary feeding-temperature trials (ME*VF/TFM). Estimates of the realized ME rates (Table 1; ME*VF/TFM) show the strong effect that *Tb* would have on daily assimilation rates in *C. collaris*, with a more than 10-fold increase in kJ/day across the 28 to 37°C trials. Furthermore, the influence of *Tb* on ME appears to have little-to-no biological significance compared to the effect of *Tb* on feeding rates and passage times in *C. collaris*.

**Table 1:** Predicted daily assimilation rates (kJ/day) of *C. collaris* across temperature treatments

	Standardized ME rates (ME/TFM)				Realized ME rates (ME*VF/TFM)			
*Tb* (°C)	C (kJ)	ME (kJ)	TFM (days)	kJ/day	C (kJ)	ME (kJ)	TFM (days)	kJ/day
28	5.28	4.65	3.54	1.31	2.11	2.04	3.54	0.58
31	5.28	3.99	2.43	1.64	4.09	2.91	2.43	1.20
34	5.28	4.30	1.70	2.53	9.38	7.31	1.70	4.30
37	5.28	4.59	1.38	3.31	13.77	11.01	1.38	7.95

Standardized metabolizable energy rates (ME/TFM) = the predicted daily assimilation rates using mean C across all trials and *Tb*-specific ME and passage rates (TFM). Realized metabolizable energy rates (ME*VF/TFM) = the predicted daily assimilation rates using *Tb*-specific voluntary feeding rates (VF) for C and *Tb*-specific ME and passage rates. TFM = adjusted (mixed model ANCOVA) mean number of days to the time of first observed marked faeces. Equations used for ME from mixed model ANCOVA fixed effects: 28°C = (0.775C + 0.399 kJ)/3.54d; 31°C = (0.775C − 0.262;kJ)/2.43d; 34°C = (0.775C − 0.042 kJ)/1.70d; 37°C = (0.775C + 0.332 kJ)/1.38d. For ME/TFM, ‘C’ = mean kJ consumed/day across all temperature treatments. For ME*VF/TFM, ‘C’ = mean kJ voluntarily consumed (VF) for each temperature treatment.

Our results indicate that predicted energy assimilation rates were greatest at 37°C. The peak in energy assimilation rates at 37°C in our study is consistent with the mean *Tb* of active *C. collaris* in intact glades (37.2°C; Brewster and Beaupre 2019). The maintenance of field-active *Tb*s that coincide with *Tb*s that maximize digestive processing rates is not uncommon in lizards (Beaupre *et al*. 1993; van Marken Lichtenbelt 1997; Angilletta 2001, Angilletta 2009). However, it is important to note that since *C. collaris* voluntary active *Tb* range is 31.2–42.6°C, our study cannot rule out the possibility that digestive performance would continue to increase with *Tb*s above 37°C (Huey 1982). Because of animal care concerns, we chose to use the species mean field-active *Tb* as the high-temperature treatment. Thus, our study is unable to determine the optimal *Tb* range for digestive performance in *C. collaris*. Regardless of the optimal *Tb* range of digestion, our study suggests that assimilation rates will decline when individuals experience *Tb*s below 37°C.

Results from this study have important implications for the conservation of *C. collaris* in the Ozarks. Encroached glades with greater shade cover and lower daily environmental heat loads (compared to intact glades) could force individuals to tolerate lower *Tb*s compared to individuals in intact glades. In turn, reduced *Tb*s in *C. collaris* can result in major declines in their energy assimilation rates (Table 1). Since *C. collaris* in encroached glades have reduced growth and reproduction, and energy available for growth and reproduction is a function of net assimilation rates (Congdon *et al*. 1982; Niewiarowski 2001; Beaupre and Zaidan 2013), our study provides a critical component to understanding factors that may drive population declines in the Ozarks. We note that a more complete understanding of the linkages among encroached glades, temperature, net assimilation rates, growth/reproduction and population declines in *C. collaris* will require data beyond those presented here. However, this study provides a robust mechanistic pathway to identifying the link between encroached glades and reduced growth/reproduction in *C. collaris*. Furthermore, this study highlights the critical data needed (e.g. *Tb* estimates and food availability) to understanding the complex patterns associated with *C. collaris* in the Ozark Mountains.

## Conclusions

Our results indicate that digestive performance is sensitive to *Tb* in *C. collaris*. Using estimates from our data, we show that predicted energy assimilation rates (Table 1) can be influenced strongly by temperature through two primary variables (feeding rates and passage time), with the greatest assimilation rates occurring at the mean surface-active *Tb* of this species (37°C). We conclude that depending on the extent that *C. collaris* experiences reduced *Tb*s in encroached glades, we would expect decreases in energy assimilation rates, and subsequent declines in growth and reproduction. Herein, we describe a novel approach to the integration of bioenergetics and conservation physiology and demonstrate the utility of using information on digestive performance to investigate conservation-based questions. We urge conservation biologists to consider bioenergetics when developing strategies to address cause-and-effect in their study systems.

## Appendix

**Appendix 1:** Linear regression analyses for the effect of C (consumption) and temperature on apparent assimilated energy (AE), metabolizable energy (ME), faeces (F) and uric acid (U) in *C. collaris.* All estimates in kJ.

	AE				ME				F				U			
Temp	slope (C)	SE	y-int.	SE	slope (C)	SE	y-int.	SE	slope (C)	SE	y-int.	SE	slope (C)	SE	y-int.	SE
28°C	0.84	0.02	2.51	1.33	0.74	0.04	2.95	2.31	0.16	0.02	-2.51	1.33	0.10	0.03	-0.44	1.40
31°C	0.85	0.04	1.45	2.14	0.73	0.07	2.91	3.70	0.15	0.04	-0.45	2.14	0.12	0.04	0.22	2.24
34°C	0.91	0.03	-0.32	1.74	0.83	0.06	1.04	3.03	0.09	0.03	1.32	1.74	0.06	0.03	3.65	1.83
37°C	0.89	0.04	0.66	1.83	0.80	0.06	1.77	3.17	0.11	0.04	-0.66	1.83	0.09	0.04	1.89	1.92
